# The Italian Taxonomic Backbone: exploiting taxonomic expertise in the country

**DOI:** 10.3897/BDJ.14.e183364

**Published:** 2026-03-05

**Authors:** Stefano Martellos, Ilaria Rosati, Martina Pulieri, Davide Raho, Matteo Conti, Marco Oliverio

**Affiliations:** 1 University of Trieste, Department of Life Sciences, Trieste, Italy University of Trieste, Department of Life Sciences Trieste Italy; 2 Centro Interuniversitario per le Biodiversità Vegetale Big Data—PLANT DATA, Department of Biological, Geo-logical and Environmental Sciences, Alma Mater Studiorum University, Bologna, Italy Centro Interuniversitario per le Biodiversità Vegetale Big Data—PLANT DATA, Department of Biological, Geo-logical and Environmental Sciences, Alma Mater Studiorum University Bologna Italy; 3 Research Institute on Terrestrial Ecosystems (IRET), National Research Council of Italy (CNR), c/o Università del Salento, Lecce, Italy Research Institute on Terrestrial Ecosystems (IRET), National Research Council of Italy (CNR), c/o Università del Salento Lecce Italy; 4 LifeWatch Italy, Lecce, Italy LifeWatch Italy Lecce Italy; 5 Department of Earth and Marine Science (DiSTeM), University of Palermo, Via Archirafi, 22, Palermo, Italy Department of Earth and Marine Science (DiSTeM), University of Palermo, Via Archirafi, 22 Palermo Italy; 6 BIOME Lab, Department of Biological, Geological and Environmental Sciences, Alma Mater Studiorum University of Bologna, Bologna, Italy BIOME Lab, Department of Biological, Geological and Environmental Sciences, Alma Mater Studiorum University of Bologna Bologna Italy; 7 Department of Biology and Biotechnologies Charles Darwin Sapienza University of Rome, Rome, Italy Department of Biology and Biotechnologies Charles Darwin Sapienza University of Rome Rome Italy; 8 Comitato Scientifico per la Fauna d'Italia, c/o Department of Biology and Biotechnologies Charles Darwin Sapienza University of Rome, Rome, Italy Comitato Scientifico per la Fauna d'Italia, c/o Department of Biology and Biotechnologies Charles Darwin Sapienza University of Rome Rome Italy

**Keywords:** checklist, fauna, flora, data interoperability, LifeWatch

## Abstract

The role of checklists ranges from documenting biodiversity in an area to supporting conservation and decision-making and stimulating further investigation. Italy has (at least partly) updated checklists for animals, vascular plants, mosses and liverworts and lichens. Thanks to the Joint Research Unit of LifeWatch Italy, several checklists are integrated into an information system aiming at a national taxonomic backbone. The system aims at integrating and standardising the data and providing a unique access point to authoritative checklists curated by Italian taxonomists. The platform has been developed customising DSpace software, which is connected to a database that manages multiple taxonomic data sources. While information for vascular plants and lichens are retrieved via REST API calls in a JSON format and structured and harmonised according to Darwin Core data schema, data on fauna are integrated into the relational database through a Darwin core-structured dataset. The checklists are updated at regular intervals thanks to the contribution from the scientific community. The Italian taxonomic backbone counts about 86000 records and its continuity and sustainability is provided by Italy’s long-term commitment to the LifeWatch ERIC. Its reliability, on the other hand, stems largely from the involvement of numerous expert taxonomists in creating and updating the checklist data. This manuscript aims at describing the Italian taxonomic backbone and the role of the scientific community which contributed to its development.

## Introduction

Checklists are a fundamental tool for documenting biodiversity within a specific area (Nimis & Martellos 2003). Modern checklists thus are pivotal as authoritative tools for conservation and decision-making and act both as a temporally contextualised inventory, as well as a stimulus for further research (Hobern et al. 2021). They also allow monitoring rare and invasive species and help ensure efficient allocation of conservation resources. However, creating a checklist demands continuous and intensive effort from taxonomists specialising in different taxonomic groups (Engel et al. 2021, Simões et al. 2025). Building a robust and reliable checklist that supports the broader scientific community requires extensive training — particularly working on scientific collections — which can take years. This is a significant issue in the context of increasing taxonomic impediment.

In Italy, fortunately, botanical and zoological taxonomic communities are still sufficiently robust to cover most taxa known to occur in the country. Furthermore, collaboration with taxonomists from other European countries can help to fill existing knowledge gaps. As a result, Italy hosts more-or-less updated checklists for nearly all organism groups, ranging from animals (Minelli et al. 1995, Ruffo and Stoch 2006) to vascular plants (Bartolucci et al. 2024, Galasso et al. 2024), mosses and liverworts (Aleffi et al. 2023), lichens (Nimis 2016) and even macro-basidiomycetes (Onofri et al. 2005), though the latter has not been updated in at least twenty years. As far as animals are concerned, the previous national checklist (Minelli et al. 1995), which was only partly updated since its original release, is currently under revision (Bologna et al. 2022).

Checklists not only offer an updated record of biodiversity, but also serve as a reliable current nomenclatural backbone. The taxonomic knowledge embedded within them is crucial for all research purposes, especially biodiversity management and conservation. Without a solid understanding of what exists in an area, biodiversity management activities cannot proceed effectively.

Thanks to the Joint Research Unit of LifeWatch Italy (hereafter JRU LW-ITA), several Italian digital checklists are being integrated into interconnected information systems that will, collectively, form the country’s taxonomic backbone. This vital national tool is sustained by the contributions of numerous taxonomists who organise and update checklist data and by data managers who promote data standardisation, aggregation and accessibility. This paper presents the knowledge base used to build the Italian Taxonomic Backbone, how it has been organised, how it is managed and its long-term sustainability.

## Data resources

The development of the country’s taxonomic backbone is based upon two major resources.

### Vascular plants and Lichenised fungi

The checklists of the land plants and fungi of Italy were all converted into digital online portals in the 2000s in the framework the *Dryades* project (Nimis et al. 2003) of the Department of Life Science, University of Trieste, which is a member of the JRU LW-ITA. Since their publication online, they have been maintained and improved and are consulted every day by hundreds of users all over Italy and abroad.

As far as the land plants are concerned, the taxonomic knowledge in the country is still wide and covers practically all the taxonomic groups. The checklists of vascular flora and lichens are updated at regular intervals; that of mosses and liverworts has been updated recently. The only one which is lagging is that of the macro-basidiomycetes, which dates back to 2004.

FlorItaly, the online database which originated from the publication of the checklists of native and alien land vascular flora, was originally released in 2008 (https://floritaly.plantdata.it). The system receives regular updates (every 6 months). Each update is first published as a scientific paper and then included in the system. Thus, the data are “frozen” at six-months intervals. Presence data are organised by administrative regions and include information on whether the presence is dubious, the presence of historical records, the alien or invasive status etc.

The checklist of the lichens of Italy was originally based on a 1993 publication (Nimis 1993), which was converted into an online information system in 2003 (Martellos 2012). Since 2003, it has evolved, with the development of several features, including digital identification keys (Nimis and Martellos 2020) into the current platform ITALIC (Martellos et al. 2023). The system aggregates checklist data, digitised herbarium occurrences, identification keys, digital images and supports advanced queries combining ecological, morphological and distributional parameters. It is continuously updated with newly-published data (https://italic.units.it).

The checklist of mosses and liverworts, published in the early 2000s (Cortini Pedrotti 2001), was updated several times, with the last update dating 2023 (Aleffi et al. 2023), but the corresponding online system has yet to be updated. Its update is scheduled for 2026.

The macro-basidiomycetes checklist is outdated, since it was published in 2005 (Onofri et al. 2005). In this case, even if the mycological community in the country is solid and quite wide, there is probably a lack of central organisation to carry out the challenging task of publishing an updated checklist.

### Fauna

The production of checklists of the Italian fauna has a long tradition. The former Checklist (Minelli et al. 1995) listed 1,812 species of protozoans and 55,656 species of Metazoa, as the product of an international team of 272 specialists from 15 countries. The Scientific Committee for the Italian Fauna is currently managing the updating of the checklist of the animal species of Italy (Bologna et al. 2022). In February 2022, lists of different taxa for more than 27,600 species have been collected and new lists are still being completed by other specialists and edited by the Steering Group. A total of more than 60,000 species of Metazoa is ultimately expected. The current version of the Checklist of the Italian fauna can be found online at https://www.lifewatchitaly.eu/en/initiatives/checklist-fauna-italia-en/checklist/.

**Figure 1.**
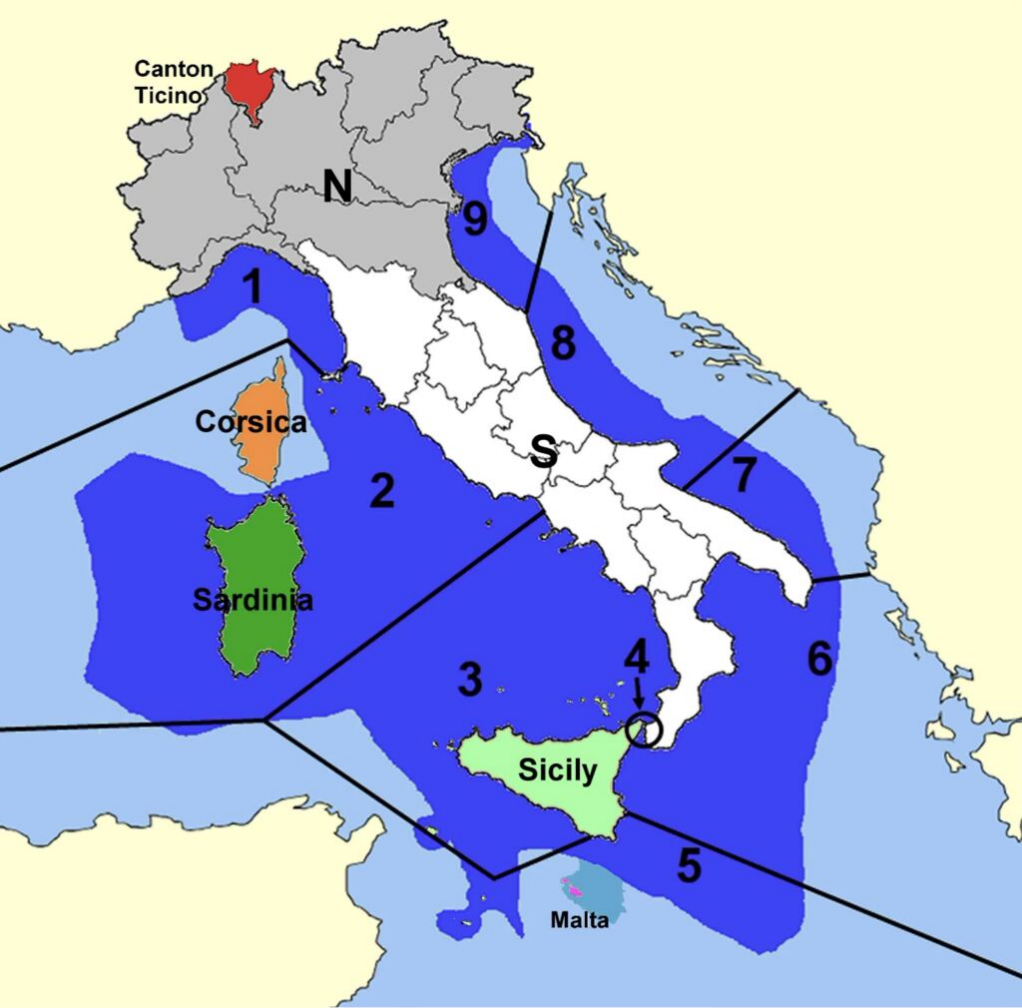
Geographical setting of the new Checklist of the Italian fauna. Terrestrial and freshwater sectors: **Canton Ticino** (*s.l.*, including Val Mesolcina, red); **N**, northern continental macro-region (graey); **S**, southern peninsular macroregion (white); island macro-region, divided in the previous checklist into **Sicily** (light green) and **Sardinia** (dark green); **Malta** (pink); **Corsica** (orange). Marine sectors, with the Italian Economic Exclusive Zone in blue: **1**, Ligurian Sea (*s.l.*); **2**, northern Tyrrhenian, including the sea around Corsica and Sardinia; **3**, southern Tyrrhenian, including the sea around northern and southern Sicily and Pantelleria Is.; **4**, Messina Strait; **5**, southern Mediterranean, including the sea around south-easternmost Sicily and the Pelagie Islands and the continental shelf around Malta (turquoise); **6**, Ionian; **7**, southern Adriatic; **8**, mid-Adriatic; **9**, northern Adriatic.

The structure of the new Checklist includes, for each species, information on the geographic range in Italy (Fig. 1), the status as endemic or alien, data on host species (for parasites) or breeding status (for birds), short taxonomic and distributional notes and reference to relevant recent literature. Data papers describing the updates to the old Checklist are being progressively published in a special section of *Biogeographia – The Journal of Integrative Biogeography* (section: *The new Checklist of the Italian Fauna*).

## Results - Data integration and standardisation in the Italian Taxonomic Backbone

**Figure 2.**
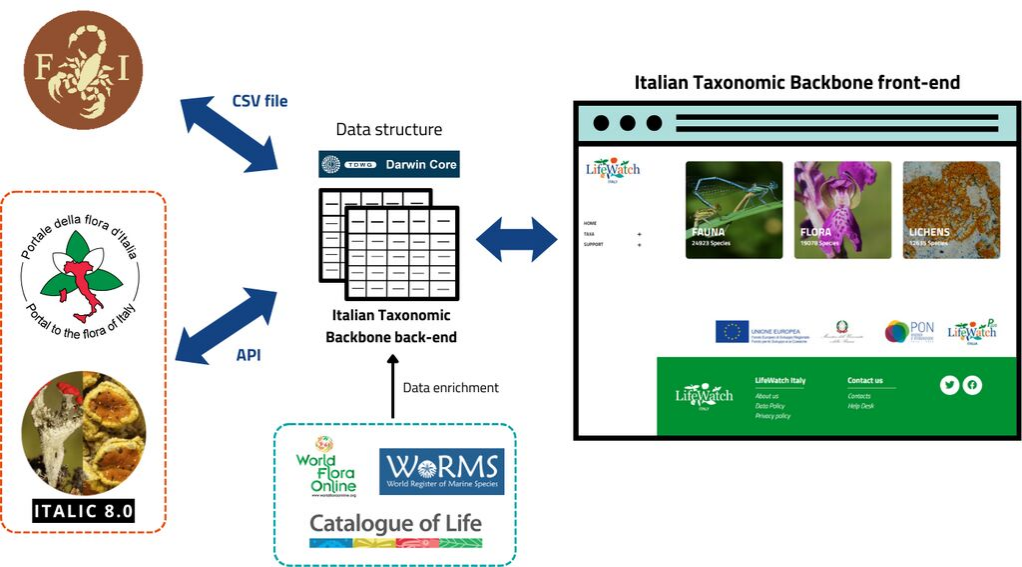
A general overview of the construction, structure and management of the Italian Taxonomic Backbone knowledge base.

The Italian Taxonomic Backbone aims to integrate, standardise and provide a centralised access point to authoritative checklists curated by Italian taxonomists (https://taxonomicbackbone.lifewatchitaly.eu). The platform has been developed through a customisation of DSpace software connected to a relational database backend that manages multiple taxonomic data sources. Taxonomic information for vascular plants and lichens are retrieved via REST API calls in a JSON format, then structured and harmonised according to Darwin Core data schema. Data on fauna are instead integrated into the relational database through a Darwin core-structured CSV file (Fig. 2). Their taxonomic classification is enriched using Global Species Databases (GSD), such us Catalogue of Life (CoL, Bánki et al. (2026); https://www.catalogueoflife.org), World Register of Marine Species (WoRMS; https://www.marinespecies.org) and World Flora Online (WFO; https://www.worldfloraonline.org). Scientific names of vascular plants, lichens and fauna are consistently linked to their equivalents in the respective GSD, to ensure taxonomic interoperability. The curation and extension of the Italian Taxonomic Backbone actively involve contributions from the scientific community. In fact, researchers and taxonomy experts can propose new taxa and submit updates and corrections for existing taxa. Each request is processed through a validation workflow managed by taxonomic group editors ensuring the validation of new taxa and the scientific reliability and consistency of the backbone with current nomenclatural standards.

**Figure 3.**
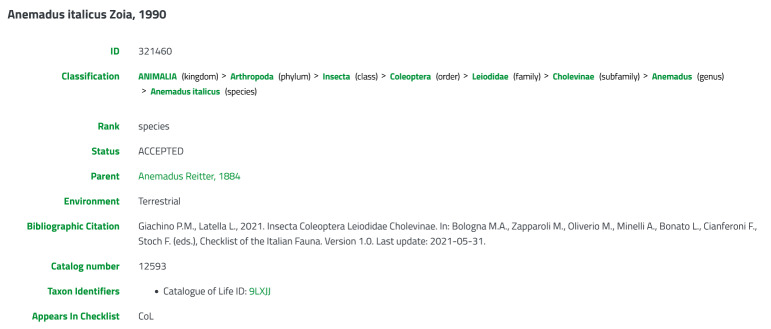
Species detail page showing all the main information of the taxon displayed.

The Italian taxonomic backbone knowledge base includes about 86,000 records. In particular, there are 7,754 accepted species names of vascular plants, 3,312 of lichens and 24,923 of fauna (Table 1). Moreover, it also includes synonyms for vascular plants and lichens and basionyms for lichens, integrating approximately 56,000 species names. All taxa come with multiple associated information within a detailed web interface (Fig. 3).

At the top of the page, the unique Italian Taxonomic Backbone ID is displayed, serving as the primary reference for the record. Immediately below, users can explore a navigable taxonomic classification, allowing them to move through higher and lower hierarchical levels. The interface also presents detailed information on synonyms and basionyms (if it exists), the status (e.g. accepted) and the taxonomic rank of the scientific name. A bibliographic citation is also provided to reference the original or authoritative source of the taxon for fauna records. Additional sections provide useful metadata, such as external identifiers from CoL, WoRMS and WFO, thereby facilitating cross-referencing and promoting taxonomic coherence, data interoperability and integration within the global biodiversity information.

All the data are distributed under the CC BY 4.0 licence.

## Discussion and Conclusions

A robust taxonomic infrastructure must be both sustainable and reliable. Consequently, the planning of the Italian Taxonomic backbone was deliberately structured to meet both these objectives.

Sustainability is assured on multiple fronts. Firstly, Italy’s long-term commitment to the LifeWatch Italy ensures continuity: as long as the ERIC persists, the service will remain operational. Secondly, at least the botanical component of the infrastructure is built entirely on open-source solutions, making it portable and deployable across any willing service centre, thus ensuring continuity even in adverse scenarios. Italy’s entry as a voting member of GBIF in October 2025 further ensures long-term sustainability. In fact, under this framework, the national taxonomic backbone is now the first check for all data published via the Italian GBIF National Node.

The checklists of vascular flora and lichens are widely used both in the country and abroad — particularly the lichen checklist — as taxonomic reference standards, receiving on average of approximately 400 and 350 unique visitors per day, respectively. Both checklists provide users with taxonomic alignment tools (Conti et al. (2021)). The new taxonomic backbone described in this manuscript has been adopted as the taxonomic reference for all databases published through the LifeWatch Data Portal (https://data.lifewatchitaly.eu/). Datasets that cannot be mobilised through the GBIF can be published via the Portal once their nomenclature has been properly verified and aligned with the national backbone. Thus, given the large and diverse user base, ensuring data reliability is a central priority in the development and management of these checklists.

Reliability, however, stems largely from the involvement of numerous expert taxonomists in creating and updating the checklist data. In Italy, as in many countries, tens of dedicated taxonomists across various specialities continue to contribute tirelessly. This extraordinary - yet often unrecognised commitment - is vital for producing quality checklists, even on narrow taxonomic groups. The phenomenon is part of the broader taxonomic impediment, a global shortage of taxonomic expertise, funding and recognition that hinders biodiversity research and conservation (Godfray 2002, Riedel et al. 2013). Since much of this work is done on a voluntary basis and not adequately rewarded by existing academic evaluation systems, it remains precarious — an issue indeed not unique to Italy. A recent global survey highlighted that taxonomists face low academic recognition, insufficient funding, publication challenges and difficulties in career progression, all of which contribute to a worrying decline in the taxonomic workforce (Engel et al. 2021). Given the fundamental role of taxonomists in biodiversity science — forming the baseline for species identification, conservation plans and ecological assessments — the role of any ERIC in biodiversity and its associated national distributed centres, particularly LifeWatch, must expand beyond infrastructure provision. It should actively foster new generations of taxonomists, mitigating the taxonomic impediment across Italy and the EU.

This can be achieved by:

- Advocating with decision-makers to revise academic evaluation metrics to fairly credit taxonomic work (Krell 2002, Löbl et al. 2023);
- Expanding positions for taxonomists in Natural History Museums, where experts can engage continuously in collection-based studies (Dupérré 2020, Engel et al. 2021).
- Promoting open-access cyber infrastructure and digitised collections, which democratise taxonomic knowledge across professional and amateur communities alike (Zauner 2009, Hedrick et al. 2020, Hardisty et al. 2022).

Community-wide infrastructure efforts like the the World Register of Marine Species (Chamberlain and Vanhoorne. 2017) demonstrated how coordinated networks of taxonomists, combined with sustained institutional support, can successfully build long-term, authoritative taxonomic backbones. Likewise, global biodiversity platforms, such as the Catalogue of Life, provide dynamic, consensus-based species checklists by integrating data from multiple specialist databases and institutions. These models underscore the key role of taxonomists — and the necessity to support them adequately.

## Figures and Tables

**Table 1.** Distribution of accepted, synonym and basionymscientific names within the Italian Taxonomic Backbone, grouped by vascular plants, lichens and fauna and taxon rank.

**Taxonomic Group**	**Taxon Rank**	**Accepted Name**	**Synonym**	**Basionym**
Vascular plants	Species	7754	11324	-
	Subspecies	3367	2803	-
Lichens	Species	3312	7385	1938
	Subspecies	51	130	27
	Variety	114	1724	280
Fauna	Species	24923	-	-
	Subspecies	2582	-	-
